# Maxillary Canine Substitution for a Central Incisor With Severe Root Resorption: A Case Report

**DOI:** 10.1155/crid/4904301

**Published:** 2025-11-14

**Authors:** Hoda Rahimi, Anil P. Ardeshna

**Affiliations:** Orthodontic Department, Rutgers School of Dental Medicine, Newark, New Jersey, USA

## Abstract

When maxillary canine emerges in an abnormal position, the so-called ectopic eruption, it can lead to issues that require a combination of surgical, orthodontic, pediatric, and restorative treatments. If the ectopic eruption causes damage to other teeth, they may need to be removed, and the remaining teeth repositioned and reshaped to achieve a functional and esthetic result. This process requires a team effort from pediatric dentists, orthodontists, radiologists, and surgeons and a knowledge of tooth anatomy, root positioning, and restorative techniques. This case report details the replacement of an impacted maxillary canine with a central incisor with a severely resorbed root.

## 1. Introduction

The permanent maxillary canine is probably the tooth most affected by ectopic eruption and transposition. The term “ectopic eruption” describes the eruption of a tooth in an abnormal position or orientation [1]. Transposition has been described as an interchange in the position of two permanent teeth within the same quadrant of the dental arch [2]. The canine is the most commonly transposed tooth, with ectopic eruption mesial to the lateral incisor or distal to the first premolar [3, 4]. Tooth transposition is a relatively rare phenomenon (0.33% prevalence) [5], more commonly observed in female patients [6–11] and in the maxilla than in the mandible [9, 12]. Unilateral canine transposition happens more frequently (79%) and is more prevalent on the left side (69%) [3–15].

Root resorption of the adjacent teeth is one of the most common and irreversible sequelae of maxillary canine impaction [16]. Root resorption is a condition associated with either a physiological or a pathological process resulting in a loss of dentine, cementum, and/or bone [17]. The detection of root resorption can greatly impact the approach to treatment. If root resorption is identified in a permanent maxillary central incisor before beginning orthodontic treatment, a decision must be made on whether to extract the affected tooth and proceed with orthodontic treatment for an impacted canine or to reposition the impacted canine away from the resorbed tooth while closing the space and reshaping the teeth.

The objectives of orthodontic treatment are to establish good static and functional occlusion and to provide pleasing facial esthetics while maintaining temporomandibular joint and periodontal health [2]. The treatment of dental transposition frequently requires a multidisciplinary approach to achieve long-term esthetic and functional results [9, 18–21].

This report describes the multidisciplinary treatment of a patient with advanced root resorption of the maxillary right permanent central incisor due to maxillary canine transposition.

## 2. Diagnosis and Etiology

A 10-year-and-5-month-old Hispanic female presented to the orthodontic clinic with an impacted maxillary canine. She was physically healthy and had no history of any medical disease or trauma.

**Figure 1:**
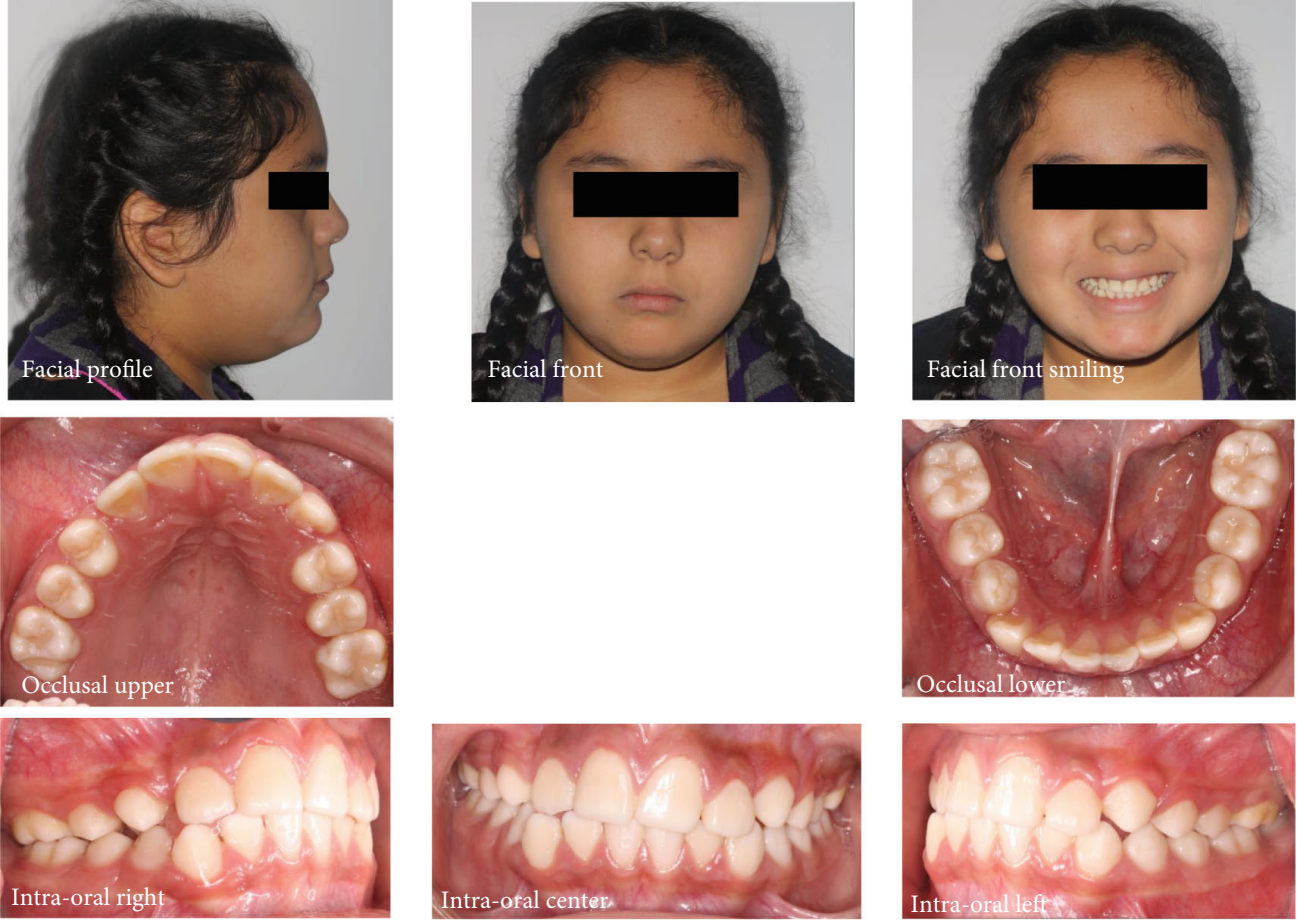
Pretreatment facial and intraoral photographs.

Facial analysis revealed a mesofacial face, a balanced profile, and obtuse nasolabial and mentolabial angles. Both the upper and lower lips were protrusive. The smile line was nonconsonant, accompanied by an anterior occlusal cant. There was a maxillary midline deviation to the right, while the mandibular midline aligned with the facial midline. Intraoral examination showed a bilateral Angle Class I molar and left canine relationship, with the maxillary right canine clinically absent. The overjet was 1 mm and overbite 2 mm with an anterior crossbite on the right. There was moderate crowding in the maxillary arch and mild crowding in the mandible arch. The Curve of Spee was accentuated on both sides. Clinical examination revealed a Class I degree of mobility of the upper right central incisor (#8). Vitality tests gave a positive response indicating a vital tooth. Palpation of the alveolar mucosa below the nasal spine revealed a bulge over the right central incisor root (Figure 1).

**Figure 2:**
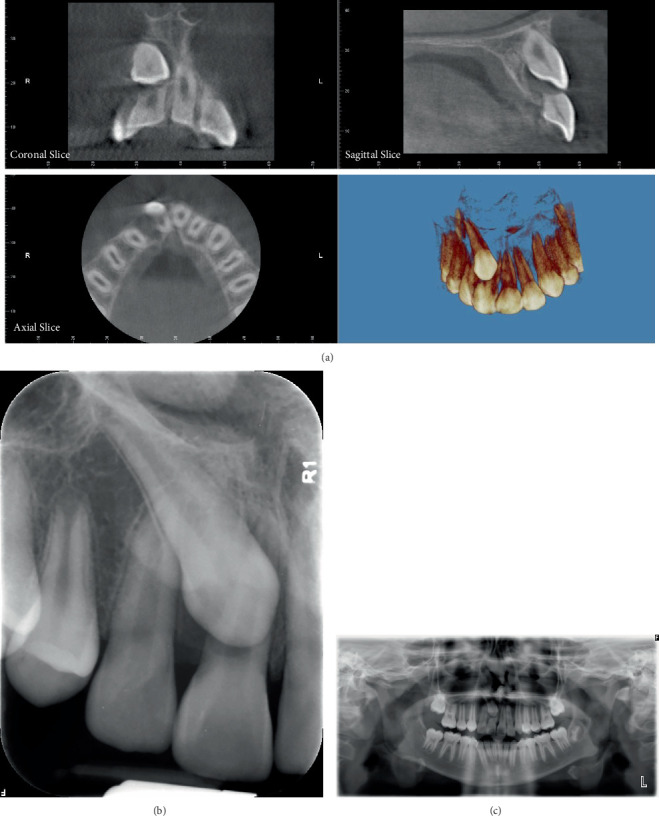
Pretreatment radiographic images demonstrating the maxillary right canine: (a) CBCT, (b) periapical X-ray, and (c) panoramic radiograph.

Both panoramic and intraoral periapical images revealed transposition of the permanent maxillary right canine [6] into the position of the permanent maxillary right central incisor (#8). CBCT images showed impaction of the maxillary right canine located in the buccal plate and at the middle one-third of the root of the adjacent teeth in the vertical direction and severe root resorption of the right central incisor. The true limits of the resorption of the incisor had an oblique pattern and were advanced mesiodistally and labiolingually. The space of the transposed canine was mostly closed by the adjacent teeth (Figure 2).

**Figure 3:**
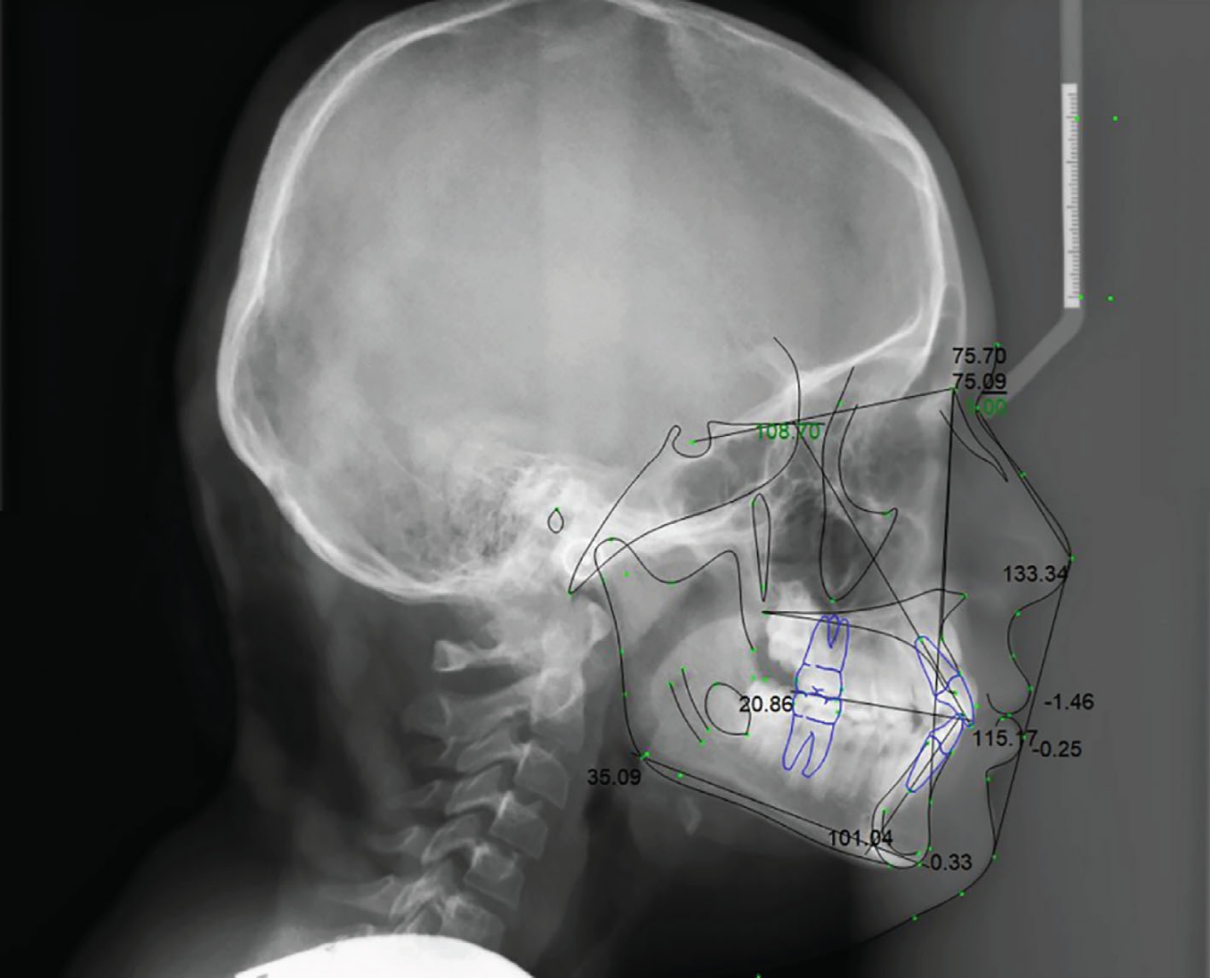
Pretreatment digitized lateral cephalometric radiograph.

Patient had a skeletal Class I relationship with a retrognathic maxilla and mandible. The maxillary incisors were both protruded and proclined, while the mandibular incisors were protruded (Figure 3 and Table 1).

### 2.1. Treatment Alternatives

Several treatment alternatives were considered, including the surgical extraction of the impacted canine only. However, this was not deemed ideal due to the poor prognosis of the right central incisor. Extracting both the impacted canine and the right central incisor, followed by replacing them with implants, was ruled out due to concerns about esthetics, function, and financial cost. No treatment was another option, but the risk of losing the central incisor due to severe root resorption necessitated intervention. The need for treatment was further emphasized by the mobility of the central incisor.

The chosen treatment involved extracting only the right central incisor, as most of its root had been resorbed, making removal necessary. The right canine, which was in the same position as the central incisor, was then moved into the space left by the extracted tooth. Once the canine was moved, it would require prosthetic restoration to function as a replacement for the missing central incisor. This approach had the advantage of utilizing the patient's own tooth, thereby eliminating the need for an artificial tooth implant or a prosthetic bridge.

### 2.2. Treatment Objectives

The following treatment objectives were planned.

1. Substitute the maxillary right canine for the right central incisor.

2. Level and align the arches.

3. Correct the maxillary midline deviation.

4. Obtain maximum intercuspation with proper overjet and overbite.

5. Improve facial esthetics and establish a functional occlusion.

6. Improve oral health.

7. Long-term stability.

### 2.3. Treatment Progress

**Figure 4:**
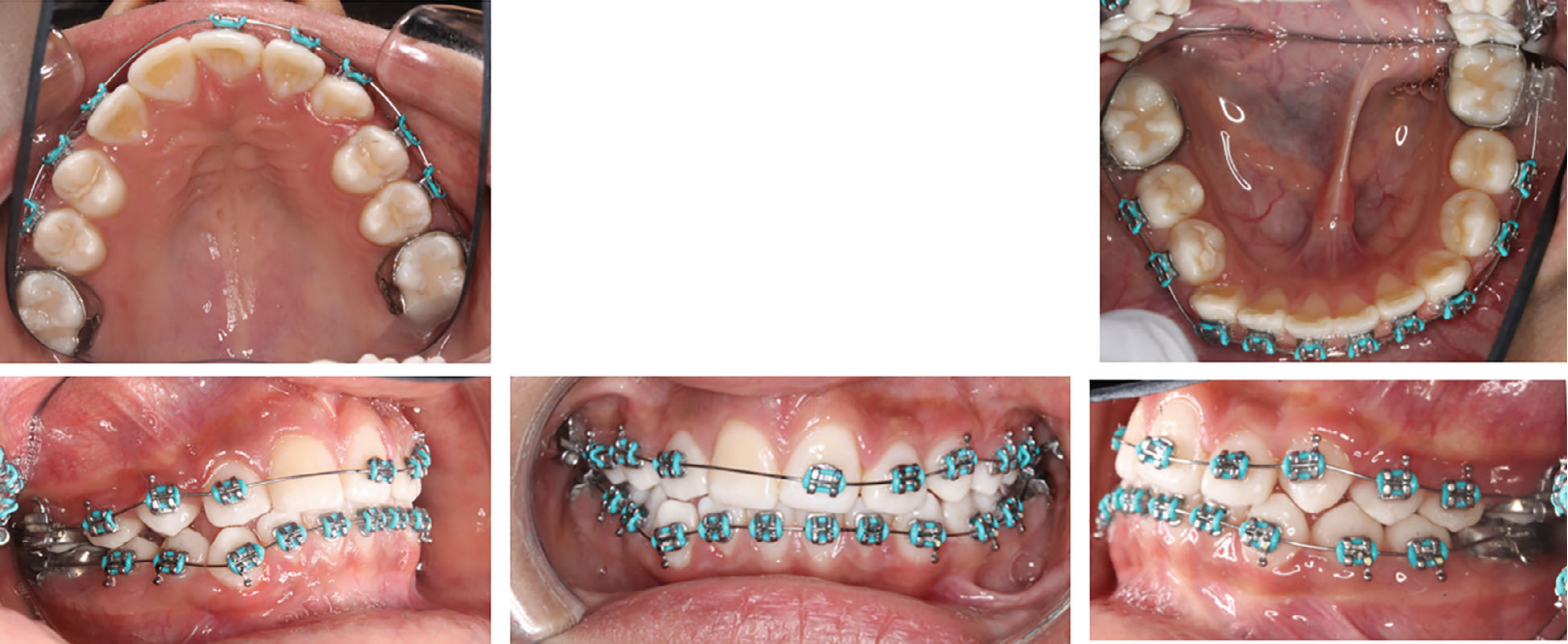
Intraoral photographs taken at the bonding appointment.

Treatment began with bonding the upper and lower arches except the upper right central incisor with MBT 0.022-in. slot brackets. 0.016-in. NiTi wires were placed (Figure 4).

**Figure 5:**
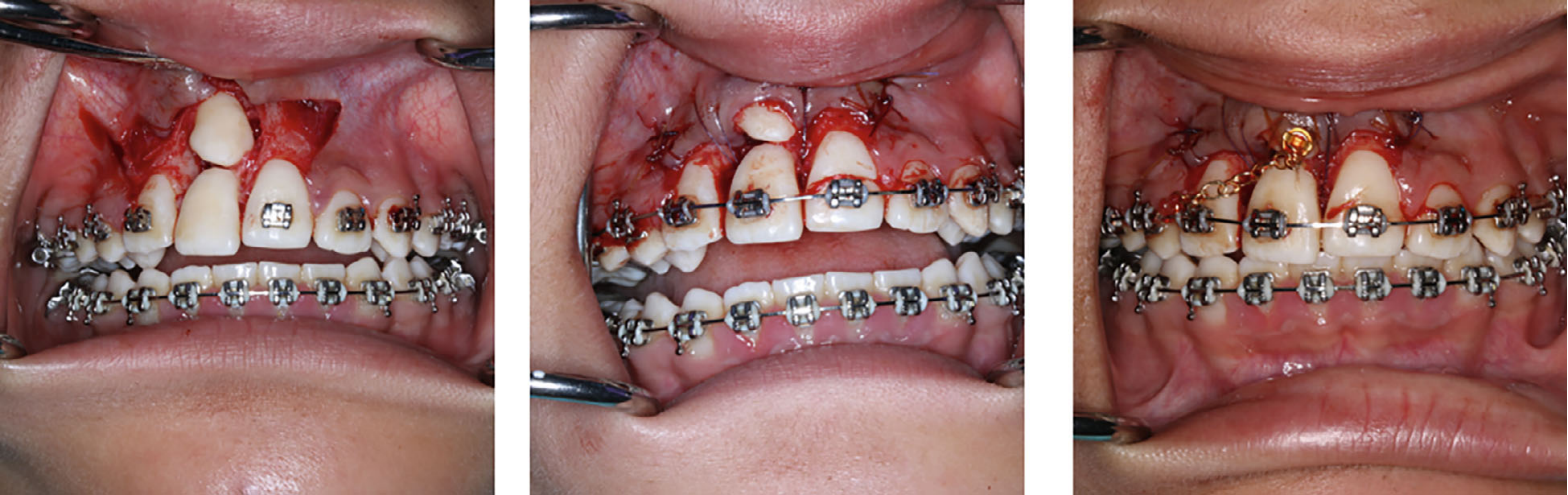
Surgical exposure of impacted canine.

Class III triangular 1/4 M elastics were used for the right side, from upper first molar to the upper and lower canine to close the open bite. After 3 months, upper and lower wires were changed to 0.016 × 0.022-in. NiTi, followed by 0.016 × 0.022-in. SS. The patient was scheduled for surgical exposure of the impacted canine and extraction of the right maxillary incisor. Following consultation with the periodontic department, it was concluded that the incisor would be retained temporarily to preserve the space, with plans to remove it later. The canine crown was surgically exposed by an apically positioned flap, and a gold chain was bonded on the buccal surface (Figure 5).

**Figure 6:**
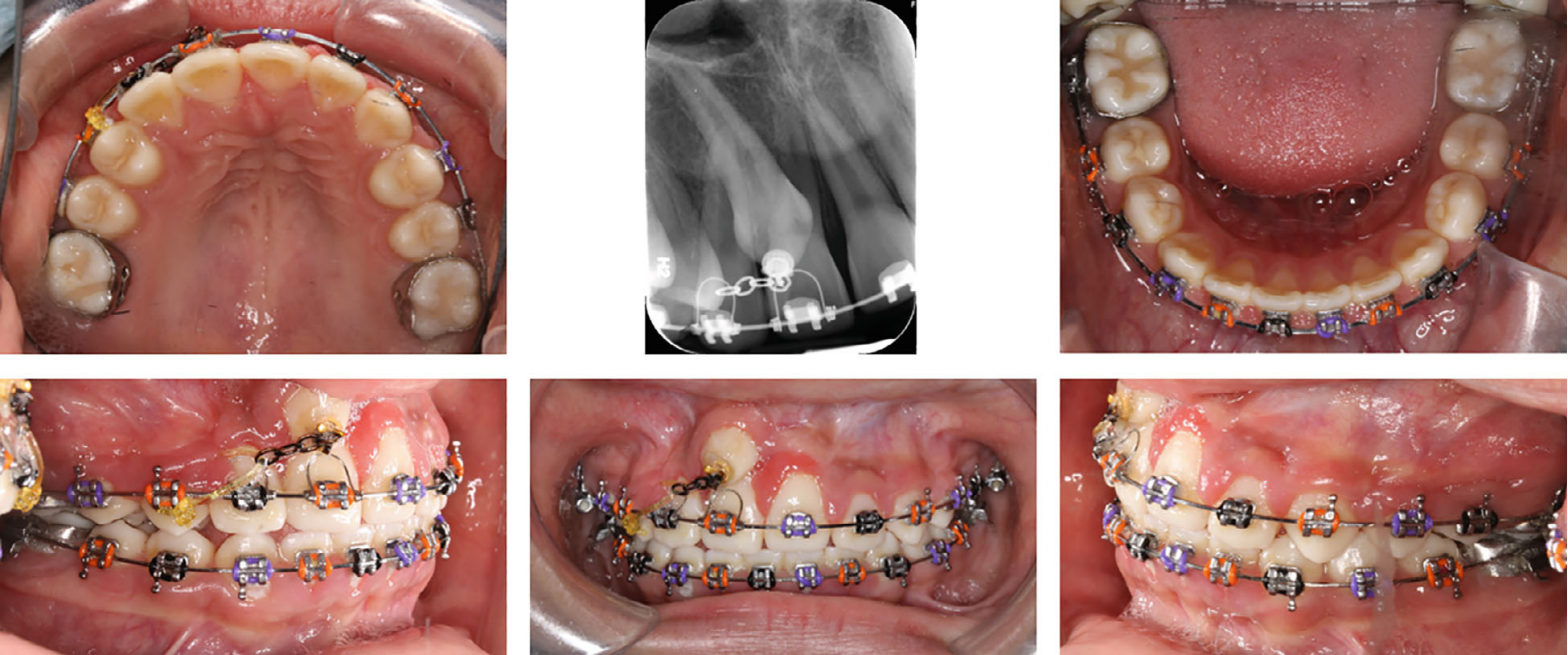
Progress with light traction force on canine.

Upper right central incisor was also bonded. Palatal root torque was adjusted for upper right central and lateral incisors by Warren Springs. A light-force elastic chain was used for traction of the canine (Figure 6).

To minimize the risk of root resorption to the lateral incisors during canine traction, light and controlled orthodontic forces were applied throughout the movement. The canine's eruption path was carefully managed using surgical exposure with an apically positioned flap, ensuring its trajectory avoided direct contact with adjacent roots. Radiographic monitoring with CBCT at multiple stages allowed early detection of any root proximity or resorptive changes. In addition, palatal root torque was applied to the adjacent teeth to further distance their roots from the erupting canine.

**Figure 7:**
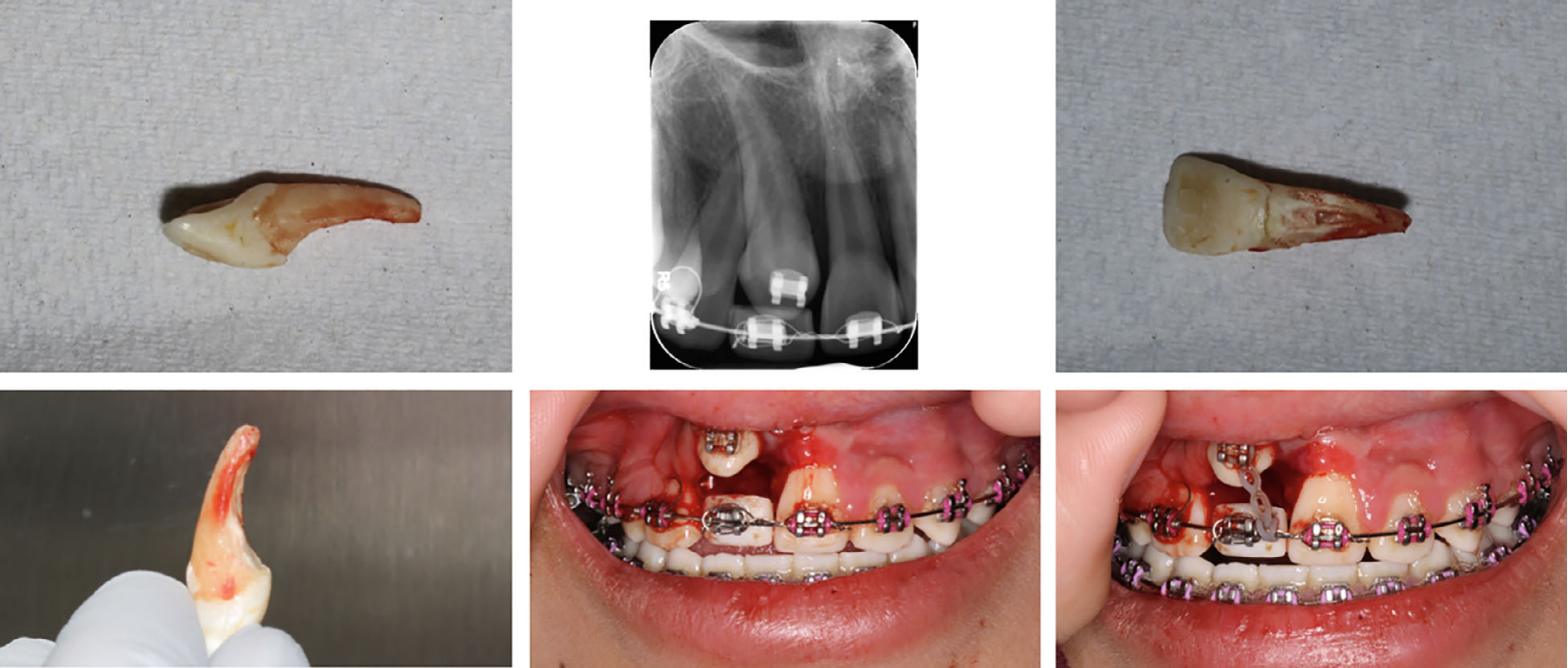
Extraction of right central incisor and using it as a temporary pontic.

Three months later, the upper right central incisor was extracted. The tooth, which had severe root resorption on the buccal aspect, was amputated, and the pulp chamber was cleaned and filled with composite to be used as a temporary pontic. It was bonded and placed back into the arch wire (Figure 7).

The lower wire was progressed to 0.017 × 0.025 SS. Once the canine erupted sufficiently, it was bracketed and the pontic removed. A 0.016-in. NiTi overlay wire was used to bring it into the arch. The maxillary arch was then leveled with a continuous arch wire, starting with 0.016-in. NiTi and working up to 0.017 × 0.025-in. NiTi wire.

**Figure 8:**
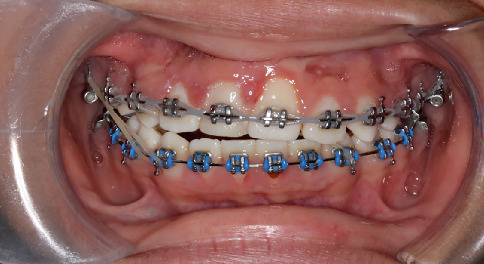
Progress in treatment.

Midline triangular elastic 1/4 M was used from upper right premolar, lower right canine, to lower left canine. Also, the patient was asked to continue wearing Class III elastic on the right side. Elastics were worn and adjusted as necessary to accommodate changes in the occlusion (Figure 8). Final finishing of the occlusion was accomplished with 0.019 × 0.025-in. stainless steel wires.

After attaining the final occlusion, the brackets were debonded. The canine was then temporarily restored with composite to resemble a central incisor. Modification of crown morphology and color was essential to obtain reasonable esthetics. During the transition to composite buildup on the substituted canine, the cosmetic nanohybrid resin 3M Filtek Supreme Ultra Universal Restorative (3M, St. Paul, Minnesota, United States) was suggested. Shaping was performed using a contoured Mylar strip for proximal adaptation. A putty silicone index from a wax-up can also be used to guide lingual and overall anatomy for greater precision. Temporary silicone paste, though not used for lingual shaping in this case, may be considered in similar treatments to enhance anatomical detail. For these reasons, cosmetic reconstruction by veneers or zirconium-based ceramic crowns accompanied by esthetic gingivectomy will be needed in the future.

For retention, Hawley retainers for the upper and lower arches were given to the patient. The overall treatment duration with the fixed brackets was 30 months.

### 2.4. Treatment Results

**Figure 9:**
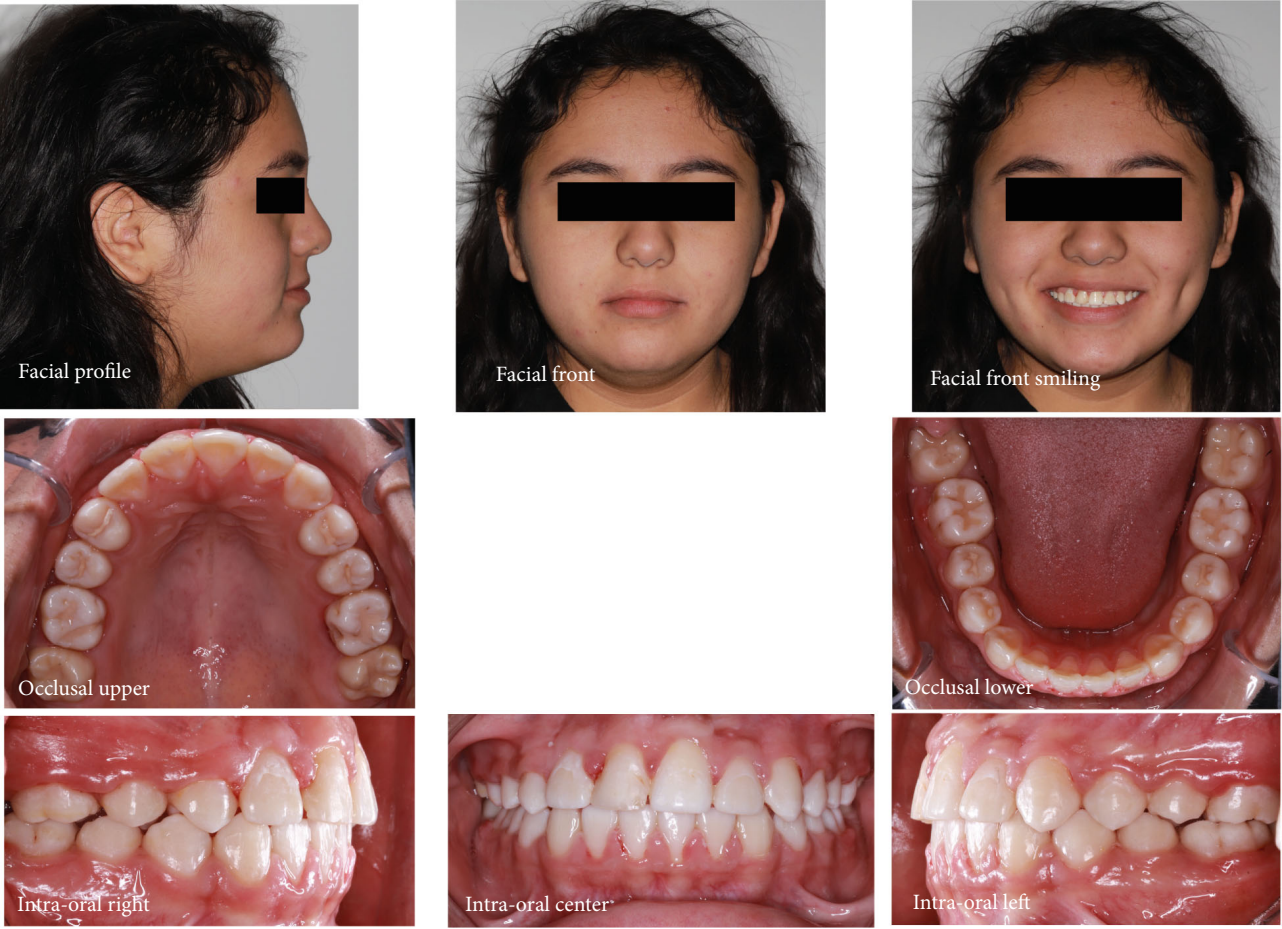
Posttreatment facial and intraoral photographs.

Posttreatment records indicate the treatment objectives were achieved. The ectopically erupting canine was effectively substituted, the crowding in both arches was resolved, and anterior crossbite was corrected. The occlusal condition after treatment achieved a functional, static occlusion with Classes I and II relationships on the left and right sides, respectively, and proper overjet and overbite. The canine was positioned in the central incisor site with careful bracket placement and torque correction to mimic the incisor's root and crown orientation. The crown was recontoured and, if needed, composite build-up was performed to harmonize the tooth's width, shape, and incisal edge, as recommended for canine substitution protocols. Functionally, the occlusion was meticulously adjusted so that the substituted canine provided centric contacts and anterior guidance during protrusive movements, simulating normal central incisor function. This was confirmed through clinical evaluation of occlusal pathways and fine-tuning restorative contours to ensure smooth protrusive function and esthetic integration. The midline deviation was improved; however, it was not fully resolved due to the inherent challenge of substituting only one maxillary central incisor and the asymmetric loss of an anterior tooth. Loss of a central incisor on one side restricts complete midline correction, as symmetrical anchorage and tooth movement are limited, resulting in a residual deviation despite best efforts to match the smile line. This is an inherent limitation in such asymmetric cases (Figure 9).

Following the correction of the transposition, the periodontal tissues appeared to be in good health with no indications of pockets or recession during clinical examination.

**Figure 10:**
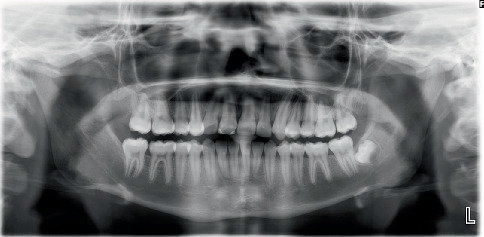
Posttreatment panoramic image.

The panoramic radiograph revealed satisfactory root alignment, except for the right upper lateral and substituted canine, which had different root anatomy compared to a central incisor (Figure 10).

**Figure 11:**
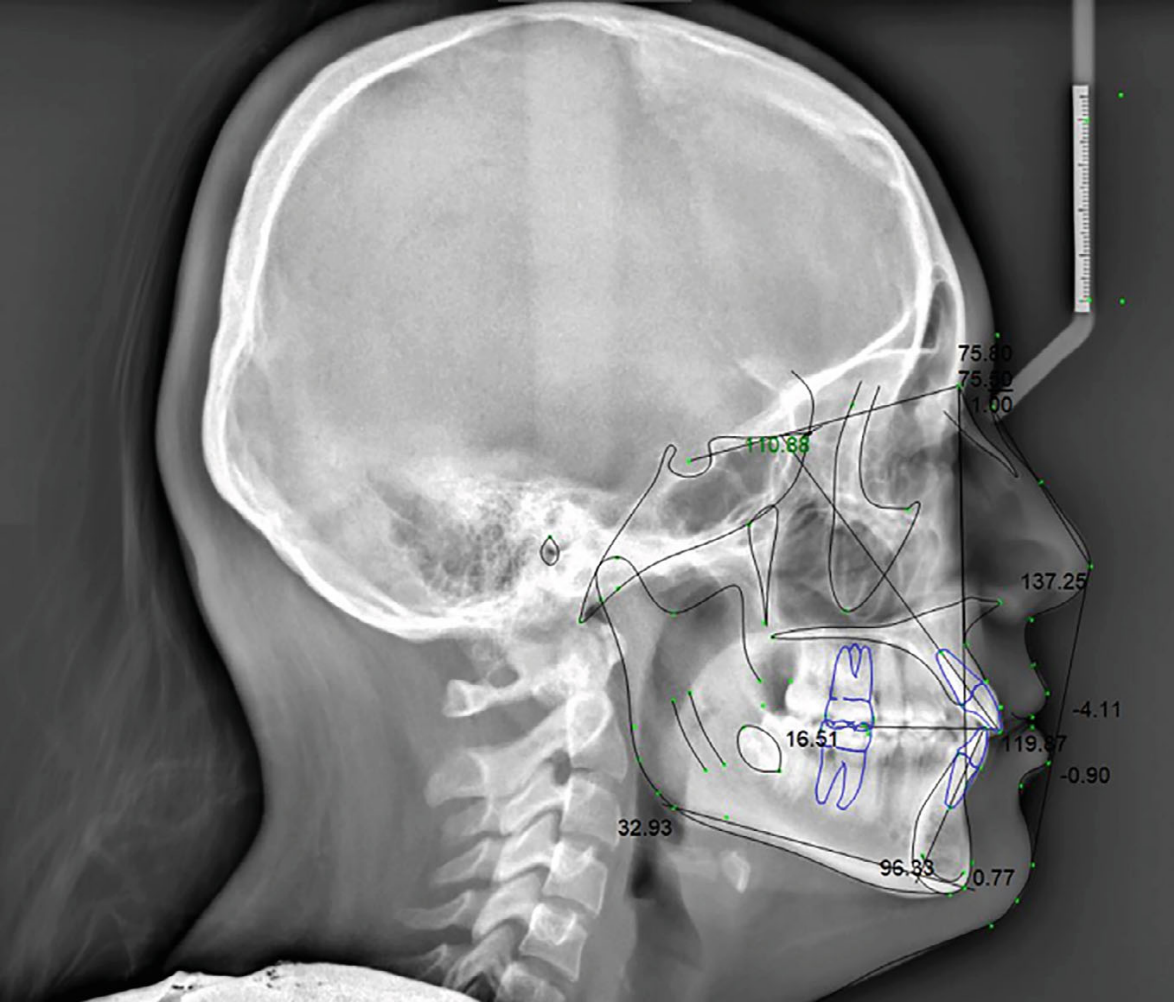
Posttreatment digitized lateral cephalometric radiograph.

**Figure 12:**
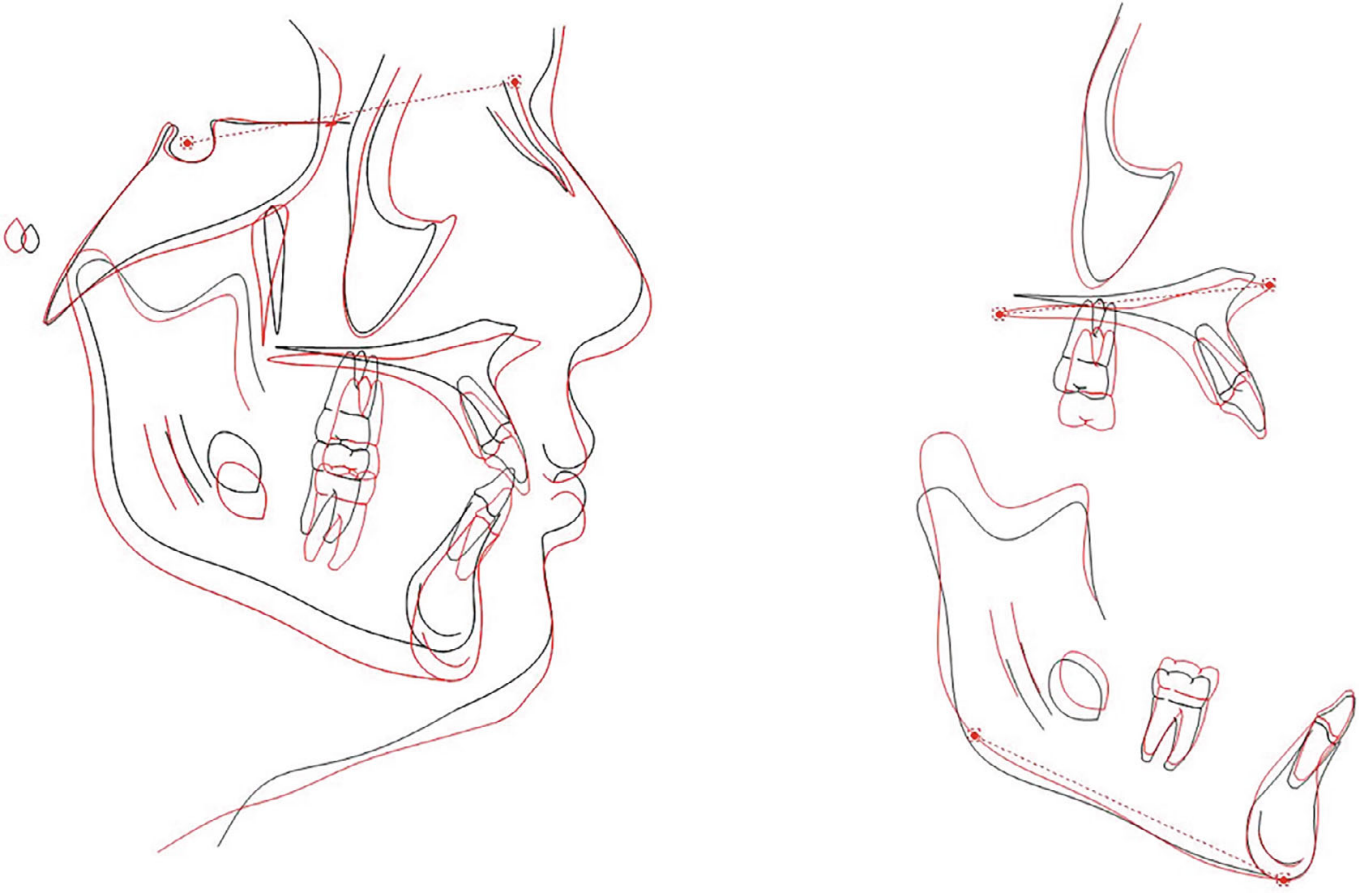
Posttreatment digitized lateral cephalometric analysis and superimposition of initial and final lateral cephalometric images.

The posttreatment lateral cephalometric radiograph and superimposition tracing illustrate both growth and the changes achieved after treatment. Improvement in occlusal plane angle, Wits appraisal, and upper and lower incisor protrusion was observed (Figures 11 and 12, Table 2).

The treatment approach was intricate and focused on resolving the transposition with minimal risk of harm to the patient. The plan of action was tailored to the individual patient's requirements, resulting in favorable cosmetic and functional outcomes with a positive long-term outlook.

## 3. Discussion

Impacted canines, when left untreated, can cause various issues, such as the displacement and loss of vitality of adjacent incisors, dental arch shortening, and formation of follicular cysts, canine ankylosis, recurrent infections, and internal or external resorption. However, an accurate diagnosis and timely intervention can impact the final treatment approach and outcome.

In the literature, tooth transposition is the positional interchange of two adjacent teeth. Transpositions are also classified as complete when both teeth have been entirely transposed or incomplete when only the crowns or the roots have interchanged positions [7, 9]. In our case, the right impacted canine has been transposed to the right incisor root and has overlapped it. Whether this situation can be classified as transposition or not is a matter of debate. Shapira and Kuftinec [9] suggested that maxillary canine to central incisor site transpositions should be classified as ectopic eruptions. This variation has been also described as a canine-incisor transposition [22, 23]. Peck S. and Peck L. [7] disputed this finding as they did not observe any distal shift in the incisors in these instances. Despite the terminology used, this type of transposition is an infrequent occurrence.

The patient described in this study showed severe root resorption in radiologic evaluations. Root resorption is usually clinically asymptomatic; yet in this case, it presented with Grade I mobility. Although a great portion of the central incisor's root was resorbed, the patient had no other symptoms associated with root resorption.

The treatment plan should be designed to the patient's circumstances, such as their age, financial situation, time, esthetics, and specific patient needs. Given our patient's age and growth stage, substituting the canine for the central incisor was the most suitable course of action. While using an incisor implant and prosthetic replacement as an alternative treatment plan may require less time, the available evidence indicates that closing the space camouflage of the canine tends to produce slightly better esthetic results, even if the difference is small [24].

The gingival level of the substituted canine was not intentionally finalized but was maintained higher than the opposite incisor to allow for future prosthetic treatment. This planned staged approach, supported by the literature [25], involves eventual esthetic crown lengthening and restorative procedures such as veneers or crowns to harmonize gingival levels and achieve optimal symmetry. This method is designed to promote predictable soft tissue healing and esthetic outcomes, especially important in growing patients where immediate surgical alteration could compromise tissue stability. The gingival zenith of the canine remains more centrally located compared to a natural central incisor, which is typically located slightly distal to the vertical axis. Adjustment of the gingival zenith position is planned at the time of the final prosthetic restoration. Methods include rotary gingival curettage, provisional restoration contour modifications, and controlled prosthetic emergence profile shaping, all designed to shift the zenith distally and create mirror symmetry with the adjacent incisor [25].

The submitted posttreatment photos indicate swelling of the interdental papillae. No follow-up photographs are currently available demonstrating complete resolution. Normally, papilla improvement occurs over time with good oral hygiene. If the tissue is not resolved with time, options like surgical papilla reconstruction or prosthetic papilla stimulation (overcontoured subgingival restorations) may be considered [25].

The literature contains a recommendation for using canines as a replacement option for maxillary incisors. According to Campbell et al., canines are a favorable choice for simulating central incisors due to their closer mesiodistal width and vertical height, as well as their compatible gingival architecture. However, to obtain satisfactory esthetic outcomes, it is necessary to make alterations to the crown's shape and color [26, 27]. Korkmaz and Yagci recommended leaving spaces to the mesial and distal of the extruded canine to acquire a width comparable to the adjacent maxillary central incisor [28]. The most suitable dental restoration procedure for our patient was postponed until it was financially feasible.

Moreover, simply extracting the incisor and waiting for the canine to naturally fill the central incisor space would not fulfill our treatment objectives. It is recommended to undergo orthodontic-surgical treatment over surgical treatment alone as it focuses on moving the impacted tooth to the correct position through extrusion [29].

This report documents the effective multidisciplinary management of impacted canine teeth through a surgical procedure, followed by orthodontic traction and alignment of the teeth in the upper central incisor site, after extraction of it. This approach eliminated the need for an implant or bridge by using the patient's own tooth, while also achieving a satisfactory level of esthetic appearance and functional occlusion. Accurate diagnosis, conservative management of the soft tissues, anchorage unit, and the direction of the orthodontic traction are important factors for the successful treatment.

## 4. Conclusions

Although substitution of maxillary canine for a central incisor is comparatively rare, the canine is a viable option as it can closely resemble a central incisor. However, precise alignment, angulation, and placement of the canine are crucial for a successful treatment outcome.

In this article, we presented a case of severe root resorption of a central incisor, managed through a multidisciplinary approach that included surgical extraction, orthodontic repositioning of the impacted canine, and its substitution for the central incisor. This treatment effectively utilized the patient's own tooth to achieve satisfactory esthetic appearance and functional occlusion without the need for implants or bridges.

## Figures and Tables

**Table 1:** Pretreatment cephalometric measurements comparing the patient's values with normative data and deviations.

**Group/measurement**	**Value**	**Norm**	**Std. dev**	**Dev. norm**
SNA (°)	75.7	82.0	3.5	−1.8
Maxillary skeletal (A-Na Perp) (mm)	−1.8	0.0	3.1	−0.6
SNB (°)	71.5	80.9	3.4	−1.7
Mand. skeletal (Pg-Na perp) (mm)	−4.1	−4.0	5.3	−0.0
ANB (°)	0.6	1.6	1.5	−0.7
Mx/Md diff (Co-Gn–Co-A) (mm)	22.8	20.4	4.0	0.6
N-ANS (perp HP) (mm)	46.2	50.0	2.4	−1.6
ALFH: AUFH	1.3	1.2	0.1	0.5
FMA (MP-FH) (°)	22.6	25.4	4.5	−0.6
MP-SN (°)	35.1	33.0	6.0	0.3
U1 most labial-A (perp to FH) (mm)	7.9	3.2	1.4	3.4
U1-SN (°)	108.7	103.2	5.5	1.2
U1-NA (mm)	7.4	4.3	2.7	1.2
IMPA (LI-MP) (°)	101.0	95.0	7.0	0.9
LI-NB (mm)	6.1	4.0	1.8	1.1
LI-NB (°)	31.2	25.3	6.0	1.0
LI protrusion (LI-APo) (mm)	5.6	2.7	1.7	1.7
Wits appraisal (mm)	−3.2	−1.0	1.0	−2.2
Palatal-occ plane (PP-OP) (°)	14.4	10.0	4.0	1.1
Lower lip to E-plane (mm)	−0.3	−2.0	2.0	0.9
Upper lip to E-plane (mm)	−4.5	−3.0	2.0	0.8

**Table 2:** Posttreatment cephalometric measurements comparing the patient's values with normative data and deviations.

**Group/measurement**	**Value**	**Norm**	**Std. dev.**	**Dev. norm**
SNA (°)	75.8	82.0	3.5	−1.8
Maxillary skeletal (A-Na perp) (mm)	−3.1	0.0	3.1	−1.0
SNB (°)	75.5	80.9	3.4	−1.6
Mand. skeletal (Pg-Na perp) (mm)	−5.5	−4.0	5.3	−0.3
ANB (°)	0.3	1.6	1.5	−0.9
Mx/Md diff (Co-Gn–Co-A) (mm)	26.8	23.6	4.0	0.8
N-ANS (perp HP) (mm)	48.9	50.0	2.4	−0.5
ALFH: AUFH	1.2	1.2	0.1	0.2
FMA (MP-FH) (°)	21.8	24.3	4.5	−0.6
MP-SN (°)	32.9	33.0	6.0	0.0
U1 most labial-A (perp to FH) (mm)	6.7	3.6	1.4	2.2
U1-SN (°)	110.9	102.7	5.5	1.5
U1-NA (mm)	3.1	4.3	2.7	−1.0
IMPA (LI-MP) (°)	96.3	95.0	7.0	0.2
LI-NB (mm)	4.3	4.0	1.8	0.2
LI-NB (°)	24.8	25.3	6.0	−0.1
LI protrusion (LI-APo) (mm)	3.8	2.7	1.7	0.6
Wits appraisal (mm)	−0.8	−1.0	1.0	0.2
Palatal-occ plane (PP-OP) (°)	9.8	10.0	4.0	−0.1
Lower lip to E-plane (mm)	−0.9	−2.0	2.0	0.6
Upper lip to E-plane (mm)	−4.1	−5.1	2	0.5

## Data Availability

The data that support the findings of this study are available on request from the corresponding author. The data are not publicly available due to privacy or ethical restrictions.
